# Potential Mechanisms for Racial and Ethnic Differences in Antimüllerian Hormone and Ovarian Reserve

**DOI:** 10.1155/2013/818912

**Published:** 2013-11-21

**Authors:** Reshef Tal, David B. Seifer

**Affiliations:** Genesis Fertility & Reproductive Medicine, Division of Reproductive Endocrinology and Infertility, Maimonides Medical Center, 1355 84th Street, Brooklyn, NY 11228, USA

## Abstract

Accumulating evidence suggests that reproductive potential and function may be different across racial and ethnic groups. Racial differences have been demonstrated in pubertal timing, infertility, outcomes after assisted reproductive technology (ART) treatment, and reproductive aging. Recently, racial differences have also been described in serum antimüllerian hormone (AMH), a sensitive biomarker of ovarian reserve, supporting the notion that ovarian reserve differs between racial/ethnic groups. The existence of such racial/ethnic differences in ovarian reserve, as reflected by AMH, may have important clinical implications for reproductive endocrinologists. However, the mechanisms which may underlie such racial differences in ovarian reserve are unclear. Various genetic factors and environmental factors such as obesity, smoking, and vitamin D deficiency which have been shown to correlate with serum AMH levels and also display significant racial/ethnic variations are discussed in this review. Improving our understanding of racial differences in ovarian reserve and their underlying causes may be essential for infertility treatment in minority women and lead to better reproductive planning, improved treatment outcomes, and timely interventions which may prolong reproductive lifespan in these women.

## 1. Introduction

Accumulating evidence suggests that reproductive potential and function may be different across racial and ethnic groups. Differences have been demonstrated in pubertal timing, infertility, outcomes after assisted reproductive technology (ART) treatment, and reproductive aging. Black females are known to initiate puberty one year earlier and to achieve pubertal milestones earlier than white females [1, 2]. While infertility affects women of all races and ethnicities, US black and Hispanic women have disproportionately greater rates of infertility than whites [3], with recent evidence suggesting that these differences have been widening [4]. Moreover, a mounting body of evidence shows racial differences in ART treatment outcomes, with black, Hispanic, and Asian races associated with significantly lower pregnancy rates and live birthrates than whites [5–9]. While environmental, socioeconomic status, behavioral, and anatomic factors are likely contributors to racial disparities in ART outcomes, significant differences still remain even when these factors are controlled for [10, 11], suggesting that genetic factors may also play a role. Racial/ethnic differences have also been described in reproductive aging, as reflected by menopausal timing [12–14] and hormonal fluctuations [15–17]. Furthermore, race may also affect the prevalence of premature menopause, as differences have been noted between white, black, and Hispanic women [18]. Thus, the longevity of ovarian function may be influenced by race/ethnicity.

These racial/ethnic differences in reproductive potential and function suggest a racial difference in ovarian reserve. Antimüllerian hormone (AMH) is widely considered a highly sensitive marker of ovarian reserve. It is a member of the transforming growth factor-*β* superfamily. AMH suppresses the cyclical recruitment of primordial follicles at the pool of growing follicles and is primarily produced by the pool of small and large preantral and early antral follicles, which are believed to serve as a proxy for the number of primordial follicles in the ovary [19, 20]. It has been suggested that AMH may be the most accurate biomarker of ovarian aging and offer several advantages over traditional biomarkers of ovarian reserve [19]. Compared with other hormonal markers of reproductive aging, AMH begins to gradually decline earlier in life [19, 21, 22], and its levels are not influenced by menstrual cycle timing, hormonal contraceptives, or pregnancy [19, 23–25]. 

Seifer et al. reported a significant difference in the mean level of AMH as a function of race or ethnicity [26]. They analyzed changes in AMH in a racially diverse, multicenter cohort study of HIV-infected women and high-risk seronegative women enrolled in the Women's Interagency HIV Study (WIHS). AMH levels were assessed at two time points in the study (median age: 37.5 years and 43.3 years). After controlling for age, BMI, smoking, and HIV status, black women demonstrated average AMH values that were 25.2% lower over time than those in whites (*P* = 0.037) [26]. In addition, AMH levels in Hispanic women were 24.6% lower over time than those in white women in the adjusted analysis, but this difference did not reach statistical significance (*P* = 0.063).This study provided the first biochemical evidence of racial differences in ovarian reserve, as measured by AMH. These findings have been recently corroborated by Gleicher et al. who reported that black women show a significantly greater age-related decline in AMH over time compared with white women [27]. Since the existence of racial/ethnic differences in ovarian reserve (as reflected by AMH) is expected to have important clinical implications for reproductive planning and infertility treatment of minority women, this review aims to shed light on and discuss possible underlying mechanisms for such racial and ethnic differences in AMH. While AMH is a sensitive marker of ovarian reserve, it is important to consider general limitations of currently available AMH assays when interpreting studies on AMH. Both the DSL and GenII AMH assays have been shown to exhibit significant within-subjects sample variability, likely related to instability of AMH under certain storage and assay conditions [28, 29]. A more robust AMH assay is expected to become available soon and should resolve these issues of AMH sample reproducibility in the future.

## 2. Genetic Factors 

During fetal life, the primordial follicle pool is formed and increases initially to a peak of 6 to 7 million oocytes at 20 wk gestation [30]. Hereafter, the primordial follicle pool declines dramatically until there are approximately 1 million oocytes within the ovaries at birth [31]. Constant recruitment of primordial follicles into the growing follicle pool takes place throughout a woman's life, which is referred to as initial recruitment. By the time of puberty approximately 500,000 follicles remain, declining to 10,000–50,000 by the late 30s; during reproductive years, ongoing growth of follicles into antral stage and loss of follicles due to atresia lead to a gradual decrease in the number of oocytes, with eventual exhaustion of the follicle pool and menopause as the final result [30–32]. There is great variability in the quantity and quality of the oocyte pool, or ovarian reserve, among women. There is also wide variability in reproductive potential and the timing of reproductive events such as menarche and menopause, both of which have strong genetic heritability, based on several twin and family studies [33–35]. Ovarian aging, which leads to menopause with the exhaustion of the follicular pool, also seems to have a genetic component [36]. Therefore, it is likely that genetic factors play an important role in racial/ethnic differences in these reproductive traits and, specifically, ovarian reserve. However, very few studies have examined possible genetic associations between race/ethnicity and AMH. 

In the first genome-wide association study (GWAS), to evaluate genetic associations with hormone markers of ovarian reserve, Schuh-Huerta et al. analyzed genetic variants associated with FSH and AMH, as surrogate measures of ovarian reserve, in a multiethnic fertile population of women [37]. Their study population included 232 Caucasian and 200 African American women who aged 25–45 and were prospectively enrolled in a community-based cohort. The authors found nominal genetic variants which were associated with FSH and AMH levels in both ethnic groups. Two genetic variants marginally associated with AMH were found in Caucasian women, located upstream of the JARID2 (jumonji, AT rich interactive domain 2) gene at 6p23 [37]. JARID2 is an ortholog of the mouse jumonji gene, which encodes a DNA-binding nuclear protein and is regulator of histone methyltransferases that negatively regulates cell growth and proliferation and is expressed in both human and mouse ovaries [38]. Therefore, JARID2 could play a role in cell growth within the developing ovary. In a related study, the same group investigated genetic variants associated with ovarian reserve as measured by antral follicular count (AFC) [39]. Interestingly, of the top 16 genetic variants associated with AFC in their study, 7 were associated with AMH levels. This is not surprising, as the number of preantral and antral follicles is thought to determine serum AMH level. Importantly, none of the associated genes in these studies have known roles in ovarian function and now represent an interesting group of candidate genes for further investigation. Future studies should also determine if any of these genetic variants may be responsible for racial/ethnic differences in AMH levels.

 Gleicher et al. reported that the distribution of fragile X mental retardation (*FMR1*) genotypes correlates with serum AMH level [40]. Based on a normal range of 26–34 (median 30) CGG repeats, the authors used CGG counts on the two X chromosome alleles to define whether a genotype is normal (*norm*), heterozygous (*het*), or homozygous (*hom*). An individual was defined as *norm *when both alleles were within range, *het *by one allele outside, and *norm/low *or *norm/high*, depending on the abnormal count allele being above or below normal range. Both alleles outside range are defined as *hom*. It was found that AMH ≤ 0.8 ng/mL was significantly associated with the number of CGG repeats; every decrease by five CGG repeats in the het-norm/low group increased the likelihood of diminished ovarian reserve by 40%, while every increase by five CGG repeats in the het-norm/high group increased the risk by 50% [40]. Moreover, FMR1 genotype was found to be associated with specific ovarian aging patterns. Women with het-norm/low genotype showed high ovarian reserve when they were young, which rapidly declined with age. In contrast, the het-norm/high genotype was associated with low ovarian reserve at young age but relative preservation of ovarian reserve into older ages [41, 42]. The same investigators reported that FMR1 genotypes vary between Caucasian, African, and Asian women [43], suggesting that FMR1 genotype may be linked to racial differences in AMH. In their study, African women showed a relatively high ovarian reserve at young age, characterized by the lowest FSH, the highest AMH, and the highest oocyte yield among races; yet, as they age they demonstrate the largest decline in AMH and oocyte yield and the poorest ovarian reserve compared to Caucasian and Asian women [27]. In contrast, Asian women showed a relatively low ovarian reserve in young age but the smallest decline in AMH and disproportional preservation of ovarian reserve at older ages compared to Caucasians and African women [27]. Remarkably, African women demonstrate a preponderance of the het-norm/low FMR1 genotype, while Asian women show a preponderance of the het-norm/high genotype [27]. Thus, FMR1 genotype may account for the observed racial differences in AMH and ovarian aging patterns.

BRCA1 and BRCA2 are crucial members of the DNA double-strand break repair family of genes, and mutations in the BRCA genes are associated with risk of breast, ovarian, and other cancers [44, 45]. Women who carry mutations in the BRCA1 gene show low response to ovarian stimulation and experience earlier menopause [46, 47]. Recently, it was reported that women who are BRCA mutation carriers display significantly lower serum AMH level than noncarriers (1.22 ± 0.92 ng/mL versus 2.23 ± 1.56 ng/mL; *P* < 0.0001) [48]. These observations support the possible role of DNA double-strand break repair in maintenance of human ovarian reserve and indicate that ovarian reserve is prematurely diminished in women with BRCA1 mutations. Several studies reported on variation in the prevalence of BRCA mutation carriers in women with breast cancer among various racial/ethnic groups. In a large US study which included 1727 breast cancer female patients younger than age 65 at diagnosis, estimates of BRCA1 prevalence were the highest in Hispanic patients (3.5%), followed by non-Hispanic whites (2.2%), African Americans (1.3%), and Asian Americans (0.5%) [49]. Prevalence was found to be particularly high in young (<35 years) African American patients (16.7%) [49]. Consistent with this study, similarly lower BRCA1 mutation prevalence rates in black patients compared with white patients were reported in 2 other population-based series of patients with breast cancer [50, 51]. In contrast to data on BRCA1 mutation frequency in the female breast cancer population, these data in the general population are very limited. Therefore, large population-based studies would be needed to establish the BRCA1 mutation frequency in the general population of different racial/ethnic groups of women and determine whether the association of BRCA1 mutation with diminished ovarian reserve and AMH may play a role in racial/ethnic differences in AMH (see Table 1).

## 3. Environmental Factors

Various environmental and lifestyle factors have been associated with serum AMH levels and may be implicated in racial/ethnic differences in AMH levels between women. Obesity is at epidemic proportions in the United States with recent data from the National Health and Nutrition Examination Survey showing that the combined prevalence of overweight and obesity was 64% in 2007-2008 among American women [52]. There is significant variation in obesity rates among American women according to race with blacks displaying the highest prevalence (49.6%), followed by Hispanics (43%) and whites (33%) [52]. Apart from being a known risk factor for diabetes, hypertension, cardiovascular disease, stroke, and certain cancers, overweight and obesity are also associated with poor ART outcomes in most studies [53–55], though the findings are not universal [56]. These observations suggest a possible association between obesity and lower ovarian reserve. Freeman et al. were the first to report an association between AMH levels and obesity [57]. In a cross-sectional study of AMH levels in late reproductive-age women, they found that AMH levels were 65% lower in obese women (BMI ≥ 30 kg/m^2^) compared to nonobese women (BMI ≤ 30 kg/m^2^) (0.016 ng/mL and 0.046 ng/mL, resp.). In their longitudinal analysis of a subgroup, obese women had significantly lower mean AMH levels over the 8-year interval compared to the nonobese women, corroborating the cross-sectional study results [57]. Consistent with these findings, in a study that was conducted to examine the impact of oral contraceptives on serum AMH levels by obesity status in reproductive-age women, it was found that AMH levels were 34% lower in the obese group compared to normal BMI women (2.9 ± 2.1 versus 4.4 ± 1.8 ng/mL, resp.) [58]. More recently, Buyuk et al. found a similar negative association between AMH and BMI among infertile women with diminished ovarian reserve, with mean random serum AMH levels being 33% lower in overweight and obese women compared with normal weight women [59]. However, in this study the investigators did not find an association between AMH and BMI in women with normal ovarian reserve [59].

The mechanisms by which obesity may influence ovarian function and AMH are unclear. One possible mechanism is lipotoxic effects on granulosa cells. It was recently demonstrated that mice fed a high-fat diet exhibit increased anovulation and decreased fertilization rates, concomitant with increased lipid accumulation, endoplasmic reticulum stress, mitochondrial dysfunction, and apoptosis in granulosa and cumulus cells [60]. In the same study, signs of lipotoxicity were observed in the follicular fluid of obese women undergoing controlled ovarian stimulation compared with normal weight women [60]. These findings support the notion that obesity has a detrimental effect on ovarian reserve, which is reflected by lower AMH. However, Su et al. reported that while AMH was lower in obese compared to normal weight late reproductive age women, no difference was found in antral follicle count, suggesting that the decrease in AMH level seen in obese women results from a physiologic process other than reduced ovarian reserve [61]. It is plausible that hormone metabolism, sequestration, or clearance may be altered in obese women. Consistent with this hypothesis, adiponectin, which is secreted from white adipose tissue and its serum levels are decreased in obese women [62], has been shown to modulate ovarian steroidogenesis in conjunction with insulin and gonadotropins [63]. Moreover, in a recent study by Merhi et al., the follicular fluid levels of leptin, another adipocytokine which is increased in obese women, were found to positively correlate with BMI and to suppress AMH and AMH receptor II gene expression in both cumulus and mural granulosa cells through the JAK2/STAT3 pathway [64]. Further studies are warranted to establish the mechanisms by which obesity decreases serum AMH levels and whether this reduction is reflective of reduced ovarian reserve or not.

Serum AMH concentration has been demonstrated to positively correlate with serum 25-hydroxyvitamin D [25(OH) D] levels [65, 66], suggesting that vitamin D deficiency is associated with lower ovarian reserve. In addition, Coney et al. found that vitamin D levels are lower in African American compared with white women [67]. In their study, black women had lower median levels of serum 25(OH)D compared with white women (27.3 nmol/L versus 52.4 nmol/L; *P* < 0.001), and 98% of black women had serum levels of 25(OH)D below 50 nmol/L compared with 45% of white women. Of note, the differences between the racial groups in the levels of 25(OH)D persisted despite adjustments for body weight, percentage body fat, and BMI [67]. These data suggest that vitamin D deficiency could account for racial differences in AMH between black and white women and that this effect is independent of BMI. Other than greater BMI, possible causes of lower vitamin D levels in black women include deeper skin pigmentation and decreased exposure to sunlight. Vitamin D may lead to increased AMH levels by a direct mechanism, as it was shown in a prostate cancer cell line that the AMH gene promoter has a vitamin D response element which binds the vitamin D-receptor complex, resulting in upregulation of AMH gene expression [68]. Further molecular investigations should elucidate the mechanism by which vitamin D may affect AMH production in granulosa cells. Given the global epidemic of vitamin D insufficiency, especially in black women [69], the observations of a relationship between AMH and vitamin D suggest that vitamin D supplementation may improve fertility outcome and minimize racial differences in ovarian reserve. Future studies are warranted to investigate these possibilities.

Multiple epidemiologic studies have reported that cigarette smoking leads to reduced ovarian function and fertility and an earlier age at menopause, suggesting that smoking impairs ovarian reserve [70]. Indeed, Sowers et al. recently demonstrated that women who were smokers had an earlier age at menopause and a more rapid decline in AMH levels, suggesting that smoking may lead to either fewer oocytes or an earlier decline in oocyte number [71]. In agreement with this study, Plante et al. found that active smoking but not former or passive smoking was associated with decreased AMH values in late reproductive age and perimenopausal women [72]. The authors suggested that smoking may directly cause depletion of antral follicles but not primordial follicles, such that smoking cessation may permit repopulation of the growing follicular pool and normalization of AMH. Similar association between smoking and AMH was also recently reported by Schuh-Huerta et al. [37]. It is well-known that smoking status varies across racial groups. In a study of women's health across the nation (SWAN) which examined vasomotor symptoms longitudinally in a multiethnic sample of US women undergoing the perimenopausal transition, African Americans had the highest rate of active smoking (24.6%), followed by Hispanics (16.7%), whites (16.6%), Japanese (12.9%), and Chinese (1.6%) [73]. Variations in smoking status among racial/ethnic groups may thus be potentially responsible for racial differences noted in ovarian reserve, as reflected by AMH.

The mechanism of tobacco's toxic effect on the ovary is unclear but may be due to effects on oocyte quantity [74], oocyte quality, or disruption of endocrine function [75, 76]. Animal studies suggest that polycyclic aromatic hydrocarbons (known carcinogens in cigarette smoke) cause oocyte destruction in mice [77, 78]. In addition, increased levels of nicotine metabolites and cigarette carcinogens have been noted in ovarian follicular fluid of active and passive smokers, indicating that toxic constituents of cigarette smoke including nicotine and cadmium have access to the follicular environment and could affect ovarian function [79–81]. Specifically, cotinine, a major metabolite of nicotine, was shown to accumulate in the nucleus and cytoplasm of granulosa cells [79], and nicotine has been shown to induce granulosa cell apoptosis [82], providing a possible explanation for reduced AMH levels observed in smokers.

In summary, accumulating evidence suggests that significant racial differences exist in ovarian reserve and reproductive aging. While multiple genetic and environmental factors may underlie these observed racial differences in AMH, additional investigation is needed to determine their relative contribution to the time course of reproductive function and ovarian reserve. Further genome-wide association studies and well-designed longitudinal studies are expected to identify more underlying genetic factors and further increase our knowledge of the extent of reproductive aging differences across racial and ethnic groups. Improving our understanding of racial differences in ovarian reserve and their underlying causes may be essential for infertility treatment in minority women and lead to better reproductive planning, improved treatment outcomes, and timely interventions which may prolong reproductive lifespan in these women.

## Figures and Tables

**Table 1:** Potential factors and associated mechanisms underlying racial/ethnic differences in serum antimüllerian hormone (AMH) levels.

Factors	Nature of association with AMH	Potential mechanism/s
Genetic factors		
JARID2 gene	Marginal association with serum AMH level in genome-wide association studies [37, 39]	JARID2 negatively regulates cell growth and proliferation and is expressed in both human and mouse ovaries [38]
FMR1 genotype	AMH ≤ 0.8 ng/mL was significantly associated with the number of CGG repeats [40]	Theoretical altered FMR1 gene expression
BRCA1 mutation	BRCA1 mutation carriers display significantly lower serum AMH levels [48]	Loss of BRCA1 increases DNA double-strand breaks in human and mouse oocytes and is associated with reduced oocyte survival in mice [48]

Environmental factors		
Obesity	Inverse correlation between BMI and serum AMH [57–59, 61]	Lipotoxic effects on granulosa cells [60] Leptin decreases AMH gene expression in cumulus and granulosa cells [64] Adiponectin modulates ovarian steroidogenesis [63]
Smoking	Smoking is inversely correlated with AMH [37, 71, 72]	Polycyclic aromatic hydrocarbons cause oocyte destruction in mice [77, 78] Nicotine and/or its metabolites accumulate in granulosa cells and induce their apoptosis [79, 82] Cigarette smoke metabolites are associated with follicular oxidative stress [75]
Vitamin D deficiency	Decreased serum vitamin D levels are associated with lower serum AMH levels [65, 66]	Vitamin D-receptor complex binds the vitamin D response element on the AMH gene promoter resulting in upregulation of AMH gene expression [68]

JARID2: jumonji AT rich interactive domain 2; FMR1: fragile X mental retardation; BRCA1: breast cancer 1; AMH: antim$\ddot{\text{u}}$llerian hormone.
